# Trinuclear Ni^II^-Ln^III^-Ni^II^ Complexes with Schiff Base Ligands: Synthesis, Structure, and Magnetic Properties

**DOI:** 10.3390/molecules25102280

**Published:** 2020-05-12

**Authors:** Anastasia N. Georgopoulou, Michael Pissas, Vassilis Psycharis, Yiannis Sanakis, Catherine P. Raptopoulou

**Affiliations:** Institute of Nanoscience and Nanotechnology, NCSR “Demokritos”, Aghia Paraskevi, 15310 Athens, Greece; natgeorgopoulou@hotmail.com (A.N.G.); m.pissas@inn.demokritos.gr (M.P.); v.psycharis@inn.demokritos.gr (V.P.); i.sanakis@inn.demokritos.gr (Y.S.)

**Keywords:** heterotrinuclear complexes, nickel, lanthanides, Schiff base, magnetic properties, crystal structure

## Abstract

The reaction of the Schiff base ligand *o*-OH-C_6_H_4_-CH=N-C(CH_2_OH)_3_, H_4_L, with Ni(O_2_CMe)_2_·4H_2_O and lanthanide nitrate salts in a 4:2:1 ratio lead to the formation of the trinuclear complexes [Ni_2_Ln(H_3_L)_4_(O_2_CMe)_2_](NO_3_) (Ln = Sm (**1**), Eu (**2**), Gd (**3**), Tb (**4**)). The complex cations contain the strictly linear Ni^II^-Ln^III^-Ni^II^ moiety. The central Ln^III^ ion is bridged to each of the terminal Ni^II^ ions through two deprotonated phenolato groups from two different ligands. Each terminal Ni^II^ ion is bound to two ligands in distorted octahedral N_2_O_4_ environment. The central lanthanide ion is coordinated to four phenolato oxygen atoms from the four ligands, and four carboxylato oxygen atoms from two acetates which are bound in the bidentate chelate mode. The lattice structure of complex **4** consists of two interpenetrating, supramolecular diamond like lattices formed through hydrogen bonds among neighboring trinuclear clusters. The magnetic properties of **1**–**4** were studied. For **3** the best fit of the magnetic susceptibility and isothermal *M*(*H*) data gave *J*_NiGd_ = +0.42 cm^−1^, *D* = +2.95 cm^−1^ with g_Ni_ = g_Gd_ = 1.98. The ferromagnetic nature of the intramolecular Ni···Gd interaction revealed ground state of total spin *S* = 11/2. The magnetocaloric effect (MCE) parameters for **3** show that the change of the magnetic entropy (−Δ*S*_m_) reaches a maximum of 14.2 J kg^−1^ K^−1^ at 2 K. A brief literature survey of complexes containing the Ni^II^-Ln^III^-Ni^II^ moiety is discussed in terms of their structural properties.

## 1. Introduction

The nature and magnitude of the exchange interaction between a 3d metal ion and various 4f metal ions have been the subject of intense investigation in the last decades. Lanthanide ions exhibit large and, in some cases, highly anisotropic magnetic moments, which in combination with different paramagnetic metal ions have led to polynuclear complexes presenting a wide variety of magnetic properties. The combination of 3d/4f metal ions in one molecule can achieve high spin ground states (via the involvement of the 3d metal ions) along with large single-ion anisotropy (via the presence of the 4f metal ions) and can provide, in most cases, single molecule magnets (SMMs) [1,2], i.e., quantum spin systems with well-defined ground state spin *S*. SMMs are easily synthesized and modified by wet chemistry methods at low temperatures and their particular characteristics such as monodispersity, crystallinity, and nanoscale dimensions provide the possibility for potential applications in data storage, quantum computing, and molecule-based spintronics devices [3].

The interest for 3d/4f heterometallic complexes arose rapidly after the observation of ferromagnetic interactions between the metal ions in a Cu_2_Gd complex by Gatteschi et al. [4]. Thereafter, synthetic strategies were developed for the designed synthesis of dinuclear 3d/4f complexes by Costes [5] and other researchers [6] in order to delve into the nature and magnitude of the magnetic interactions between the metal ions. It was concluded that in CuLn and NiLn dinuclear complexes the magnetic exchange interaction is antiferromagnetic for the 4f^1–5^ ions and ferromagnetic for the 4f^7–11^ ions [6]. Trinuclear 3d/4f/3d complexes have been also reported and some have been found to display SMM properties [7,8,9]. Considering the Ni^II^-Ln^III^-Ni^II^ complexes, a handful of examples has been reported with magnetic behavior ranging from ferro- to antiferromagnetic depending on the nature of the lanthanide ion. The ligands used are mainly Schiff bases based on salicylaldehyde, *o*-vanillin and tripodal, dipodal aliphatic and aromatic amines; few examples are reported with other type of ligands, e.g., *β*-diketonato or thiourea derivatives.

The structurally characterized Ni^II^-Ln^III^-Ni^II^ complexes can be classified in different ways, based on the type of ligands, the number and type of bridging atoms, and the topological characteristics of the trinuclear moiety. A survey of the literature reveals that the known Ni^II^-Ln^III^-Ni^II^ complexes show some common characteristics, irrespective of the type of ligands used. In the majority of the cases the bridging between the terminal Ni^II^ ions and the central Ln^III^ ion is provided by two or three phenolato oxygen atoms of the ligands. In few cases, carboxylato ligands can contribute to the bridging. The Ni^II^-Ln^III^-Ni^II^ moiety can be linear or bent with angles in the wide range 54–180° [10].

**Scheme 1:**
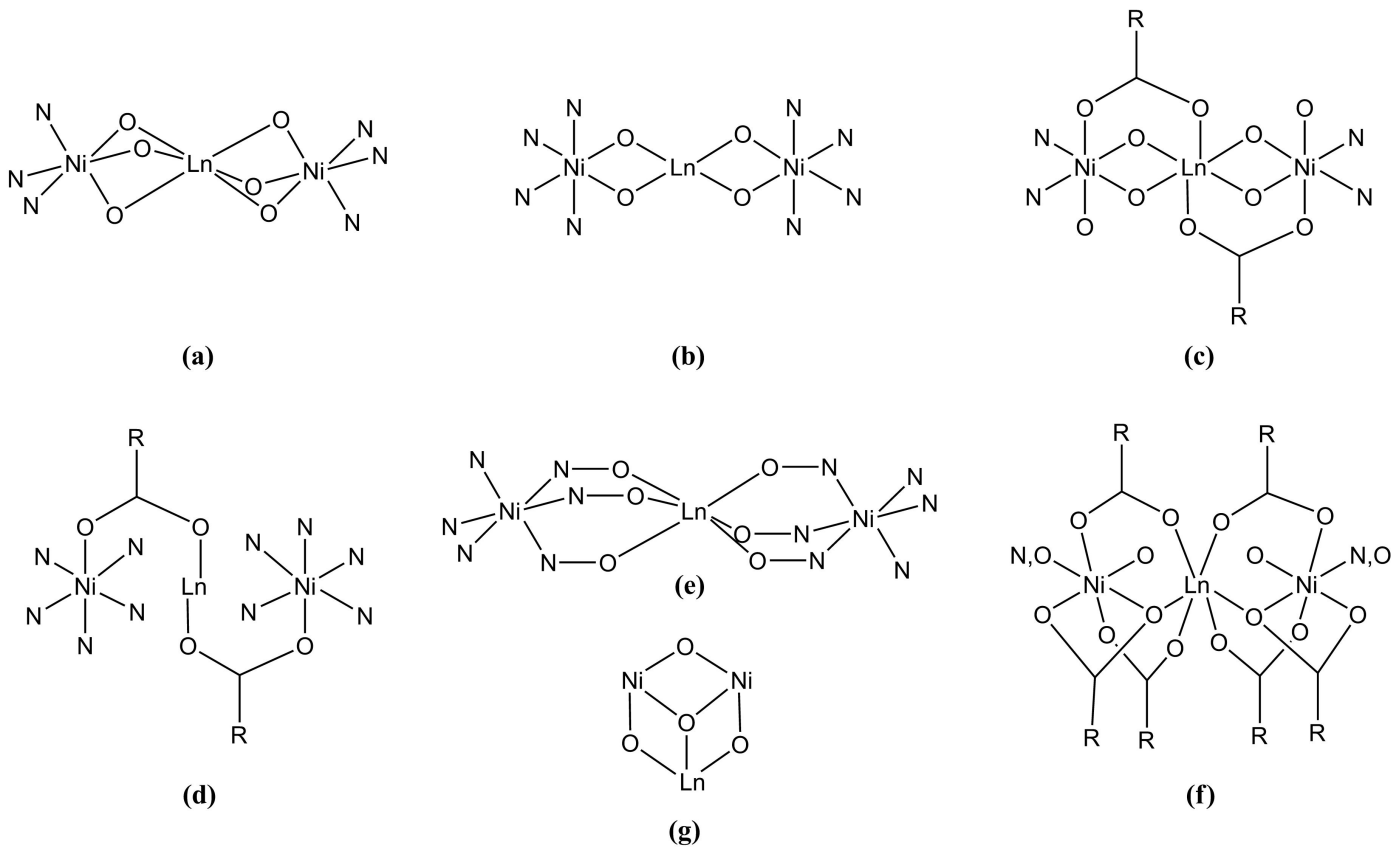
The crystallographically established metal cores in Ni_2_Ln complexes found in refs. [11,12,13,14,15,16,17,18,19,20,21,22,23,24] (**a**), [25,26,27,28,29,30,31,32,33,34] (**b**), [35,36,37,38] (**c**), [39] (**d**), [40,41,42] (**e**), (**f**) [43], and (**g**) [44,45].

Complexes [(NiL^1^)_2_Ln](ClO_4_) (H_3_L^1^ = (S)P[N(Me)N=CH-C_6_H_3_-*2*-OH-*3*-OMe]_3_, Ln^III^ = La-Er, except Pm) [11,12], [(NiL^2^)_2_Gd](NO_3_) (H_3_L^2^ = 6,6′-((1*E*)-((2-(((*E*)-(2-hydroxy-3-methoxybenzylidene)amino)methyl)-2-methylpropane-1,3-diyl)bis(azanylylidene))bis(methanyl ylidene))bis(2-methoxyphenol) [13], [(NiL^3^)_2_Ln](NO_3_) (Ln^III^ = Gd, Tb, Dy) and [(NiL^3^)_2_Dy](ClO_4_) (H_3_L^3^ = 6,6′,6″-((1*E*,1′*E*)((nitrilotris(ethane-2,1-diyl))tris(azanylylidene))tris(methanylylidene))tris(2-methoxyphenol) [14], [(NiL^4^)_2_Ln(H_2_O)_1/0_](ClO_4_) (H_3_L^4^ = 6,6′,6″-((1,4,7-triazonane-1,4,7-triyl)tris(methyl ene))tris(2-methoxy-3-methylphenol), Ln^III^ = Y, La, Ce-Lu, except Pr, Pm, Yb) [15], [(NiL^5^)Tb](ClO_4_) (H_3_L^5^ = 6,6′,6″-((1,4,7-triazonane-1,4,7-triyl)tris(methylene)) tris(2,3-dimethylphenol) [16], [(NiL^6^_3_)_2_Ln](NO_3_) (HL^6^ = (*Z*)-2-methoxy-6-((phenylimino)methyl)phenol, Ln^III^ = La, Pr, Gd, Tb) [17,18], and [{Ni{L^7^)_1.5_}_2_Ln(OH)] (H_2_L^7^ = 7,7′-(ethane-1,1-diyl)bis(quinolin-8-ol), Ln^III^ = Eu, Tb, Gd) [19] contain a linear Ni-Ln-Ni moiety (Ni-Ln-Ni ≈ 165–180°) in which the metal ions are bridged through three phenolato oxygen atoms from three ligands (Scheme 1a). Complexes [(NiL^8^)_2_Ln(NO_3_)] (H_3_L^8^ = 2,2′-((1*E*)-((2-(((*E*)-(2-hydroxybenzylidene) amino)methyl)-2-methylpropane-1,3-diyl)bis(azanylylidene))bis(methanylylidene)) diphenol, Ln^III^ = Gd, Eu, Tb, Dy) [20,21], [(NiL^9^)_2_Ln(solv)_x_)](ClO_4_) (H_3_L^9^ = 2,2′-(((2-(((2-hydroxybenzyl)amino)methyl)-2-methylpropane-1,3-diyl)bis(azanediyl))bis(me thylene))diphenol, Ln^III^ = La, Pr, Nd, Gd, Dy, Ho, Er, Yb; solv = H_2_O/MeOH/EtOH; x = 1,2) [22], [(NiL^10^)_2_Ln(MeCN)_2_](ClO_4_) (H_3_L^10^ = 2,2′,2″-((1,4,7-triazonane-1,4,7-triyl)tris(methylenen))triphenol, Ln^III^ = La, Nd, Gd, Dy, Yb) [23] and [{Ni(L^11^)_3_}_2_La(L^11^)] (HL^11^ = quinoline-8-ol) [24] also contain a Ni-Ln-Ni moiety (Ni-Ln-Ni ≈ 129–158°) in which the metal ions are bridged through three phenolato oxygen atoms (Scheme 1a).

Each of the terminal Ni^II^ ions in the above complexes [11,12,13,14,15,16,17,18,19,20,21,22,23,24] is coordinated to three phenolato oxygen atoms and three nitrogen atoms, consisting of in situ formed (NiL)^−^ metalloligand. The coordination geometry around the Ni^II^ ions is distorted octahedral except in the case of [{Ni{L^7^)_1.5_}_2_Ln(OH)] [19] in which the N_3_O_3_ atoms create a regular trigonal-antiprismatic geometry around the Ni^II^ ions. The N_3_O_3_ coordination around each Ni^II^ ion in the above complexes [11,12,13,14,15,16,17,18,19,20,21,22,23,24] imposes chirality thus, each Ni^II^ ion is chiral with either a Δ or a Λ configuration due to the screw coordination arrangement of the achiral ligands around the metal ion. When two chiral molecules associate, both homochiral (Δ-Δ or Λ-Λ) and heterochiral (Δ-Λ) pairs are possible. Since the above complexes [11,12,13,14,15,16,17,18,19,20,21,22,23,24] crystallize in centrosymmetric space groups, molecules with Δ-Δ and Λ-Λ pairs coexist in the crystals to form racemic crystals. The Ln^III^ ions present coordination modes which vary from distorted octahedral and quasi trigonal prism for O_6_ coordination [14,15,16,22,23], capped trigonal prism and capped octahedron for O_7_ coordination [14,15,22], square antiprism and bicapped octahedron for O_8_ and NO_7_ coordination [20,21,23,24], tricapped trigonal prism for O_9_ coordination [19], and distorted icosahedron for O_12_ coordination [11,12,17,18].

Complexes [{Ni(HL^12^)_2_}_2_La(NO_3_)](NO_3_)_2_ (H_2_L^12^ = (*Z*)-2-(((2-(hydroxymethyl) phenyl)imino)methyl)-6-methoxyphenol [25], [{Ni(H_3_L^13^)_2_}_2_Ln](NO_3_)_3_ (H_4_L^13^ = (*Z*)-2-((2-hydroxy-3-(hydroxymethyl)benzylidene)amino)-2-methylpropane-1,3-diol, Ln^III^ = Gd, Tb, Dy, Ho) [26], [{NiL^14^(H_2_O)}_2_Ln(H_2_O)](trif)_3_ (H_2_L^14^ = 6,6′-((1*E*,1′*E*)-((2,2-dimethylpropane-1,3-diyl)bis(azanylylidene))bis(methanylylidene))bis(2-methoxyphe nol), Ln^III^ = Gd, Eu, trif = triflate anion) [27], [(NiL^15^)_2_Ln(NO_3_)_2_(MeOH)_4_](NO_3_) (H_2_L^15^ = 1,1′-(pyridine-2,6-diyl)bis(butane-1,3-dione), Ln^III^ = La, Ce, Pr, Nd, Sm, Eu, Gd), [(NiL^15^)_2_Ln(NO_3_)_2_(H_2_O)_2_(MeOH)_2_](NO_3_) (Ln^III^ = Sm, Eu, Gd), [(NiL^15^)_2_Ln(NO_3_)_3_(MeOH)_4_] (Ln^III^ = Gd, Tb, Dy), and [(NiL^15^)_2_Ln(NO_3_)_2_(H_2_O)(MeOH)_3_](NO_3_) (Ln^III^ = Ho, Er, Tm, Yb, Lu) [28] contain linear Ni-Ln-Ni moiety (Ni-Ln-Ni ≈ 169–180°) in which the central lanthanide ion is bridged to the two terminal Ni^II^ ions through two phenolato oxygen atoms (Scheme 1b). The coordination environment around the Ni^II^ ions consists of two imino nitrogen atoms and four oxygen atoms [25,26], or six oxygen atoms [28] and is distorted octahedral. In the case of [{NiL^14^(H_2_O)}_2_Ln(H_2_O)](trif)_3_ the Ni^II^ ion is five-coordinate, linked to the N_2_O_2_ site of the ligand in the equatorial plane and to a water molecule in the apical position.

Complexes [(NiL^16^)_2_Ln(MeOH)](NO_3_) (H_3_L^16^ = 2,2′,2″-(((nitrilotris(ethane-2,1-diyl))tris(azanediyl))tris(methylene)triphenol, all Ln^III^ except Ce and Pm), [(NiL^16^)_2_Ln(MeOH)](ClO_4_) (Ln^III^ = La, Pr, Nd, Sm, Gd, Dy, Ho, Er) [29,30], [(NiL^17^)_2_Ln)](ClO_4_) (H_3_L^17^ = 6,6′,6″-(((nitrilotris(ethane-2,1-diyl))tris(azanediyl))tris (methylene))tris(2-methoxyphenol), Ln^III^ = Gd, Dy) [31], [(NiL^18^)_2_Ln(NO_3_)_2_](NO_3_) (H_2_L^18^ = 6,6′-((1*E*,1′*E*)-(ethane-1,2-diylbis(azanylylidene))bis (methanylylidene)) bis(2-ethoxyphenol), Ln^III^ = La, Ce) [32], [(NiL^19^)_2_Ce(NO_3_)_2_](NO_3_) (H_2_L^19^ = 6,6′-((1*E*,1′*E*)-(ethane-1,2-diylbis(azanylylidene))bis(methanylylidene))bis(2methoxyphe nol)) [33] and [(NiL^20^)_2_Ce(NO_3_)_3_] (H_2_L^20^ = 2,2′-((1*E*,1′*E*)-(propane-1,3-diylbis(azanylylidene))bis(ethan-1-yl-1-ylidene))dephenol) [34], contain a bent Ni-Ln-Ni moiety with angles in the range 139–144° [29,30], 113° [31], 123° [34], and 62–68° [32,33]. The central lanthanide ion is bridged to the two terminal Ni^II^ ions through two oxygen atoms (Scheme 1b). The coordination geometry around the Ni^II^ ions is distorted octahedral with N_4_O_2_ chromophore [29,30,31] and square planar with N_2_O_2_ chromophore [32,33,34]. The coordination geometry around the central Ln^III^ ion is described as flattened pentagonal bipyramid for O_7_ coordination [29,30], distorted dodecahedron [31], and square antiprism for O_8_ coordination [26], sphenocorona [25], hexadecahedron and distorted tetradecahedron for O_10_ and N_2_O_8_ coordination [28,34], and distorted icosahedron for O_12_ coordination [32,33].

Complexes [(NiL^21^)_2_Ln(O_2_CMe)_2_(MeOH)_2_](NO_3_) (H_2_L^21^ = 6,6′-((1*E*,1′*E*)-(propane-1,3-diylbis(azanylylidene)) bis(methanylylidene))bis(4-bromo-2-methoxy phenol, Ln^III^ = La, Nd, Ce, Pr) [35,36], [(NiL^22^)_2_Ln(O_2_CMe)_3_(MeOH)_x_] (H_2_L^22^ = 3,3′-(pyridine-2,6-diyl)bis(1-phenylpropane-1,3-dione, Ln^III^ = Gd, Ce; x = 2 or 3), [(NiL^22^)_2_Ln(O_2_CPh)_3_(solv)_x_] (Ln^III^ = Gd, solv = MeOH, x = 2; Ce, solv = MeOH/H_2_O x = 2) [37] and [(NiL^23^)_2_Pr(O_2_CMe)_3_(MeOH)_2_] (H_2_L^23^ = *N*^2^,*N*^6^-bis(diethylcarbamo thioyl)pyridine-2,6-dicarboxamide) [38] contain a linear Ni-Ln-Ni moiety (Ni-Ln-Ni ≈ 175–180°) in which the central Ln^III^ ion is bridged to each of the terminal Ni^II^ ions through two oxygen atoms of the ligands and one carboxylato group (Scheme 1c). Each Ni^II^ ion in [(NiL^21^)_2_Ln(O_2_CMe)_2_(MeOH)_2_](NO_3_) is coordinated to the N_2_O_2_ donor atoms of the ligands, one bridging acetato oxygen atom and one MeOH in distorted octahedron. Each Ni^II^ ion in [(NiL^22^)_2_Ln(O_2_CMe)_3_(MeOH)_x_], [(NiL^22^)_2_Ln(O_2_CPh)_3_(solv)_x_] and [(NiL^23^)_2_Pr(O_2_CMe)_3_(MeOH)_2_] presents distorted octahedral coordination derived by the O,O or O,S atoms of the ligands, one carboxylate oxygen and one MeOH. The geometry around the Ln^III^ ions is described as hula-hoop HH-9 for N_2_O_7_ coordination [37], pentagonal antiprismatic for O_10_ coordination [35,36], staggered dodecahedron SDD-10 for N_2_O_8_ coordination [37], and double-capped square antiprism for N_2_O_8_ coordination [38].

Complexes [{Ni(H_2_L^24^)(tren)_2_}_2_Ln](NO_3_)_3_ (H_4_L^24^ = 3,3′-((1E,1′E)-((((2-aminoethyl)azanediyl)bis(ethane-2,1-diyl))bis(azanylylidene))bis(methanylylidene)) bis(2-hydroxybenzoic acid; Ln^III^ = Gd, Dy, Er, Lu; tren = tris(2-aminoethyl)-amine) contain a V-shaped Ni-Ln-Ni moiety with angles 93–95° [39]. The central Ln^III^ ion is bridged with each of the terminal Ni^II^ ions through the carboxylate group of the ligands (Scheme 1d). Each of the Ni^II^ ions presents N_5_O distorted octahedral coordination and the central Ln^III^ ions shows O_8_ distorted square antiprismatic geometry.

Complexes (Me_4_N)[{(Ni(L^25^)_3_}_2_Ln(L^25^)_2_] (Ln^III^ = La, Ce, Pr, Nd, Sm) and (Et_4_N)_2_[{(Ni(L^25^)_3_}_2_Ln(dcnm)_2_](ClO_4_) (Ln^III^ = La, Ce) contain a ligand formed in situ via transition metal promoted nucleophilic addition of MeOH to a nitrile group of dicyanonitrosomethanide (dcnm) [40]. Both types of complexes contain bent Ni-Ln-Ni moiety with angles ~142 and ~133° respectively. The central Ln^III^ ion is bound to each of the Ni^II^ ions through three N-O groups from three ligands (Scheme 1e). The Ni^II^ ions present N_6_ distorted octhahedral coordination and the Ln^III^ ions show N_2_O_8_ coordination with geometry described as sphenocorona JSPC-10. Similar bridging mode with three oximato N-O groups is observed in the linear complexes [{Ni(L^26^)_3_}_2_Tb](NO_3_) [41] and [(Ni(HL^27^)_3_}_2_Tb](NO_3_) [42] (HL^26^ = (*Z*)-1-(pyridine-2-yl)ethenone oxime, and HL^27^ = (*E*)-*N*′-hydroxypyrimidine-2-carboximidamide) which contain two terminal Ni^II^ ion in N_6_ distorted octahedron and a central Tb^III^ ion bound to six oximato oxygen atoms.

Complexes [{Ni(piv)_3_(bpy)}_2_Ln(NO_3_)]·MeCN (Hpiv = pivalic acid, bpy = 2,2′-bipyridine, Ln^III^ = Gd, Sm) are isomorphous and contain a Ni-Ln-Ni moiety with angles ~153° in which the central Ln^III^ ion is bridged to each of the terminal Ni^II^ ions through two *syn*,*syn* pivalato groups and one μ-O carboxylate oxygen (Scheme 1f) [43]. The distorted octahedral coordination around each Ni^II^ ion consists of four carboxylato oxygen atoms and two nitrogen atoms of the bpy. Replacement of the bidentate bpy ligand with two monodentate ligands, i.e., Hpiv and MeCN, gave the isomorphous complexes [{Ni(piv)_3_(Hpiv)(MeCN)}_2_Ln(NO_3_)] (Ln^III^ = La, Pr, Sm, Eu, Gd) which contain a Ni-Ln-Ni moiety with angles ~144° [43]. The metal ions are bridged in similar fashion as above (Scheme 1f). The Ni^II^ ions show NO_5_ distorted octahedral coordination and the central Ln^III^ is bound to six carboxylato oxygen atoms and one chelate nitrato group in single-capped pentagonal bipyramid.

Complexes [Ni_2_(L28′)_3_(L28″)Ln(NO_3_)(H_2_O)](ClO_4_)_2_ (L28′ = ethoxydi(pyridine-2-yl)methanol, L28″ = di(pyridin-2-yl)methanediol, Ln^III^ = Gd, Tb) and [Ni_2_(L28′)_4_Ln(NO_3_)(H_2_O)][Ln(NO_3_)_5_](ClO_4_)_2_ (Ln^III^ = Tb, Dy, Y) consist of dications which contain triangular Ni-Ln-Ni moieties with angles ~54° [44,45]. The metal ions are linked through one μ_3_-O atom and three μ_2_-O atoms from the ligands (Scheme 1g).

The magnetic susceptibility measurements of the above Ni^II^-Ln^III^-Ni^II^ complexes reveal the presence of dominant antiferromagnetic interactions in the case of Ln^III^ = Ce, Pr, Nd, and ferromagnetic interactions for Ln^III^ = Gd, Tb, Dy, Ho, Er, and Yb. In the Ni_2_Gd complexes the presence of the isotropic Gd^III^ ion facilitated the fitting of the magnetic data and the determination of the Ni···Gd exchange coupling constant which is approximately +0.5 cm^−1^. Magnetization data vs. applied magnetic field at low temperatures revealed a ground state with total spin *S* = 11/2 in agreement with ferromagnetic interactions between two Ni^II^ (*S* = 1) and one Gd^III^ (*S* = 7/2) ions. In two cases, the magnetocaloric effect of the Ni_2_Gd complexes was determined by heat capacity and isothermal magnetization measurements yielding magnetic entropy change (−Δ*S*_m_) in the range ~12–14 Jkg^−1^K^−1^ [17,26]. In some cases, complexes with Ln^III^ = Dy, Tb showed zero-field or field-induced slow relaxation of the magnetization.

**Scheme 2:**
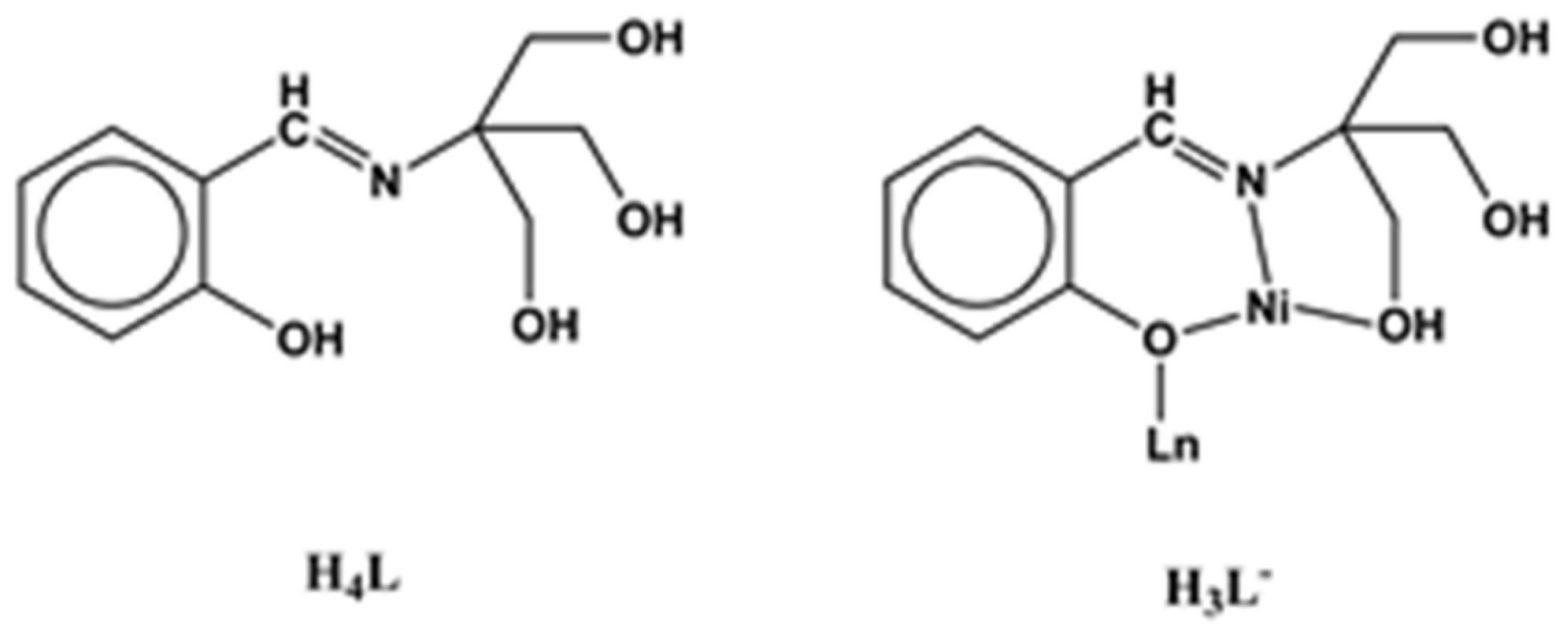
The ligand used in this work and its coordination mode in **1**–**4**.

With these considerations in mind we have explored a general reaction scheme involving Ni(O_2_CMe)_2_·4H_2_O, lanthanide nitrates, and the tetradentate Schiff base ligand H_4_L = *o*-OH-C_6_H_4_-CH=N-C(CH_2_OH)3 (Scheme 2) in an attempt to prepare oligo-/polynuclear Ni/Ln complexes for further studies of their magnetic behavior. We have isolated the trinuclear complexes of general formula [Ni_2_Ln(H_3_L)_4_(O_2_CMe)_2_](NO_3_), Ln = Sm (**1**), Eu (**2**), Gd (**3**), and Tb (**4**), containing the monoanion of H_4_L ligand. We present herein their synthesis, crystallographic characterization, and magnetic properties studies.

## 2. Results and Discussion

### 2.1. Synthesis and Spectroscopic Characterization

The reaction of two equivalents of Ni(O_2_CMe)_2_·4H_2_O with one equivalent of Ln(NO_3_)_3_·6H_2_O (Sm^III^ (**1**), Eu^III^ (**2**), Gd^III^ (**3**), Tb^III^ (**4**)) and four equivalents of H_4_L in EtOH afforded trinuclear compounds of the general formula [Ni_2_Ln(H_3_L)_4_(O_2_CMe)_2_](NO_3_) according to Equation (1). Precipitation of the microcrystalline and/or single crystal X-ray diffraction quality crystals of **1**–**4** was achieved by layering of the reaction solution with mixture of Et_2_O/*n*-hexane.

(1) $$\begin{array}{l} 2Ni{\left(O_{2}CMe\right)}_{2}\cdot {4H}_{2}O+Ln{\left(NO_{3}\right)}_{3}\cdot 6H_{2}O+4H_{4}L\overset{\mathrm{EtOH}}{\to }\\ {}[Ni_{2}Ln{\left(H_{3}L\right)}_{4}{\left(O_{2}CMe\right)}_{2}]\left(NO_{3}\right)+2HNO_{3}+2HO_{2}CMe+14H_{2}O\\ \;\;\;\;\;1–4 \end{array}$$

The identity of **1**–**4** was confirmed by single crystal X-ray crystallography, infrared spectroscopy and microanalytical techniques. Initial attempts to prepare **1**–**4** from MeOH solutions gave microcrystalline products in very low yield (only few crystals). Subsequently, we changed the reaction solvent to EtOH and we managed to increase the yield of **1**–**4** and determine the crystal structure of **4**·4EtOH·4H_2_O. The identity of **3** was confirmed by unit cell determination [46].

The IR spectra of all complexes (Figure S1) exhibit broad bands in the range 3437–3461 cm^−1^ attributed to the ν(OH) vibrations due to the presence of alkoxo groups of the ligand. The band at ~1635 cm^−1^ in the free ligand is due to ν(C=N) vibration. The shifting of this band to lower frequency (~1600 cm^−1^) in the spectra of all complexes suggests coordination of the metal ions through the imino nitrogen. The ν(C-O) stretching frequency of the phenolic oxygen of the ligand is seen at 1395 cm^−1^ and shifts to lower frequency in the spectra of all complexes, in the range 1317–1320 cm^−1^, indicating coordination to the metal ions [47]. The strong bands at ~1555 and ~1444 cm^−1^ are attributed to the *ν*_as_(CO_2_) and *ν*_s_(CO_2_) stretching vibrations of the bidentate chelate acetato ligands. The difference Δ = *ν*_as_(CO_2_) − *ν*_s_(CO_2_) is 110–114 cm^−1^ and agrees with the low difference values found in rare earth acetates with chelating coordination mode [48,49,50]. The strong band at ~1384 cm^−1^ in the spectra of all compounds is attributed to the presence of *ν*_3_(*E*′) [*ν*_d_(NO)] mode of the uncoordinated *D*_3*h*_ ionic nitrates [51].

### 2.2. Description of the Structure

**Figure 1:**
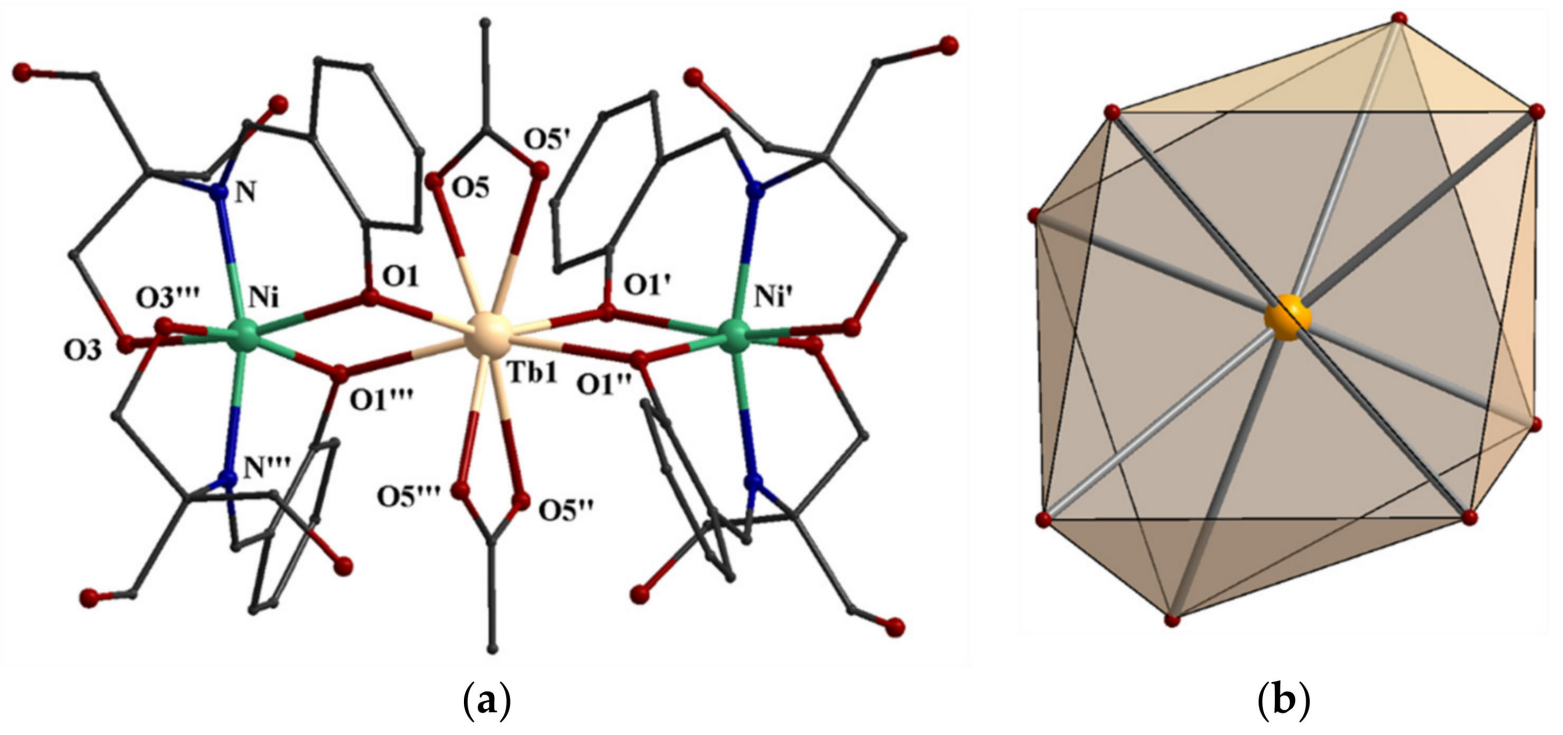
(**a**) Partially labeled plot of the cationic part of **4**. Color code: Ni^II^ green; Tb^III^ tan; O red; N blue; C grey. Symmetry operations: (′) 1.5-*x*, 0.5-*y*, *z*; (″) *x*, 0.5-*y*, 1.5-*z*; (′″) 1.5-*x*, *y*, 1.5-*z*. (**b**) The square antiprism around the Tb^III^ ion.

Compounds [Ni_2_Gd(H_3_L)_4_(O_2_CMe)_2_](NO_3_) (**3**) and [Ni_2_Tb(H_3_L)_4_(O_2_CMe)_2_](NO_3_) (**4**) are isomorphous as established by unit cell determinations. The molecular structure of **4** consists of [Ni_2_Tb(H_3_L)_4_(O_2_CMe)_2_]^+^ cations, NO_3_^−^ counteranions and solvate molecules. Compound **4** crystallizes in the centrosymmetric orthorhombic space group *Pnnn*. The asymmetric unit cell contains 1/4 of the trinuclear molecule, 1/4 of the nitrate counteranion and solvate molecules; the latter will not be discussed further. The central Tb^III^ ion resides on site symmetry 222 at (3/4, 1/4, 3/4), Wyckoff position 2d, and the terminal Ni^II^ ions reside on two-fold axis of symmetry at (3/4, y, 3/4), Wyckoff position 4i. The carbon atoms of the acetato ligands also reside on two-fold axis of symmetry at (3/4, 1/4, z), Wyckoff position 4l. The nitrate counteranion is bisected by a two-fold axis of symmetry passing through the nitrogen and one of the oxygen atoms, both reside at sites (x, 1/4, 1/4), Wyckoff position 4g; the NO_3_^−^ is disordered over two positions around site symmetry 222. Due to the symmetry described above, the angle defined by the metal ions in **4** is strictly 180° with interatomic distances Ni···Tb = 3.447 Å. The central Tb^III^ ion is linked to each of the Ni^II^ ions through two deprotonated phenolato oxygen atoms from two different ligands (Figure 1). Each terminal Ni^II^ ion is coordinated to two ligands through their phenolato oxygen, the imino nitrogen atoms and one of the protonated alkoxo groups. The coordination environment around the terminal Ni^II^ ions is distorted octahedral. The central Tb^III^ ion is coordinated to four phenolato oxygen atoms from the four ligands, and four carboxylato oxygen atoms from two acetates which are bound in the bidentate chelate mode. The eight coordination polyhedron around the Ln^III^ ion, according to the Continuous Shape Measures approach (CShM) by using the program SHAPE [52], is best described as square antiprism, SAPR-8, with the two bases formed by O(5)/O(1′)/O(5″)/O(1′″) and O(1)/O(5′)O(1″)/O(5′″), respectively. Selected bond distances are listed in Table 1.

**Figure 2:**
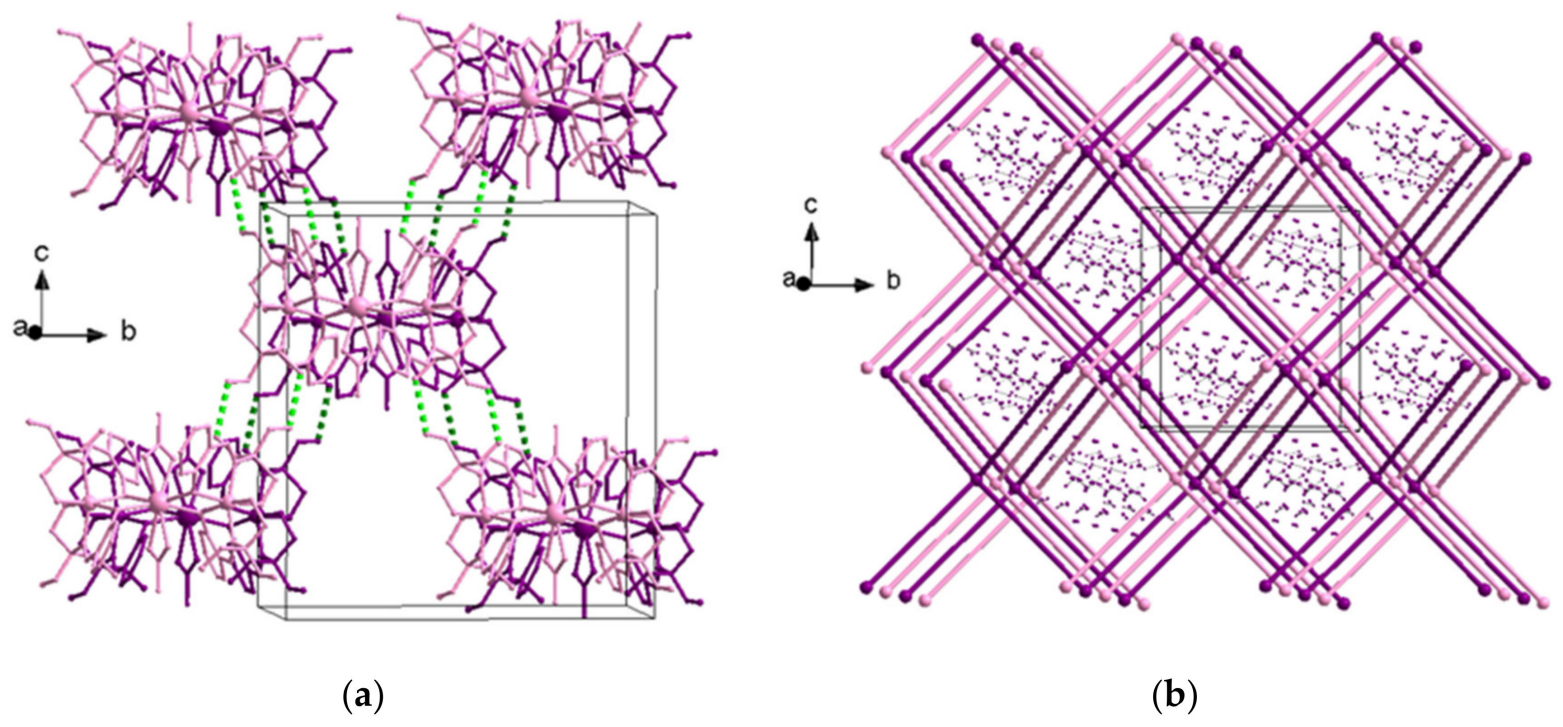
(**a**) The two 4-connected nodes of trinuclear clusters in **4**. The clusters/nodes creating one of the two interpenetrating lattices are indicated in pink color and the corresponding hydrogen bonds are shown as light green dashed lines. Those of the second lattice are indicated with violet color and the corresponding hydrogen bonds with dark green dashed lines. (**b**) A small part of the 3D supramolecular network of **4** using only the nodes in the representation consisting of two interpenetrating diamondlike lattices, indicated with pink and violet colors. In the channels formed along *a*-axis the counteranion and the solvent molecules are hosted.

The lattice structure of **4** is built due to hydrogen bonding interactions (Table 2). Each trinuclear cluster acts as a 4-connected node and is linked to four neighboring clusters through eight hydrogen bonds developed between the protonated pendant alkoxide groups of H_3_L^−^ (Figure 2a) which expands to a 3D diamondlike network. Each trinuclear cluster is translated along *a*-axis resulting in a second 3D diamondlike network interacting weakly with the first one through Van der Waals forces, and thus the final architecture of the structure consists of two interpenetrating diamond lattices (Figure 2a,b). The topology of each independent lattice is described by the 6^6^ Well point symbols or with Schläfli symbol (6,4) and the overall structure as 2-fold based on the Bratten-Robson classification scheme [53] or with topology **dia** belonging to Class **Ia**, with Translational Degree of interpenetration Z_t_ = 2 and translation along [1,0,0] (10.0634 Å) according to Blatov et al. [54]. The characteristic interlocked adamantane units of two independent interpenetrating diamondlike lattices [53] observed in the structure of compound **4** are shown in Figure S2. Schiff bases have been proved to build very interesting supramolecular structures, with the involvement of solvent molecules or not [55]. Diamondlike interpenetrating lattices, and especially those created through supramolecular interactions, have attracted the interest of researchers as it is possible to control, both the density and the pore size of the materials [56]. The center/nodes in the diamondoid lattices are at 14.386 Å apart. In channels created along the *a*-axis of compound **4**, the NO_3_^−^ counteranions and the lattice solvents (water and ethanol molecules) are hosted (Figure 2b). The channels along the *a*-axis occupy the ~38% of the total unit cell volume (with a volume of 1374.9 Å^3^ out of a total of 3642.9 Å^3^).

### 2.3. Magnetic Measurements

**Figure 3:**
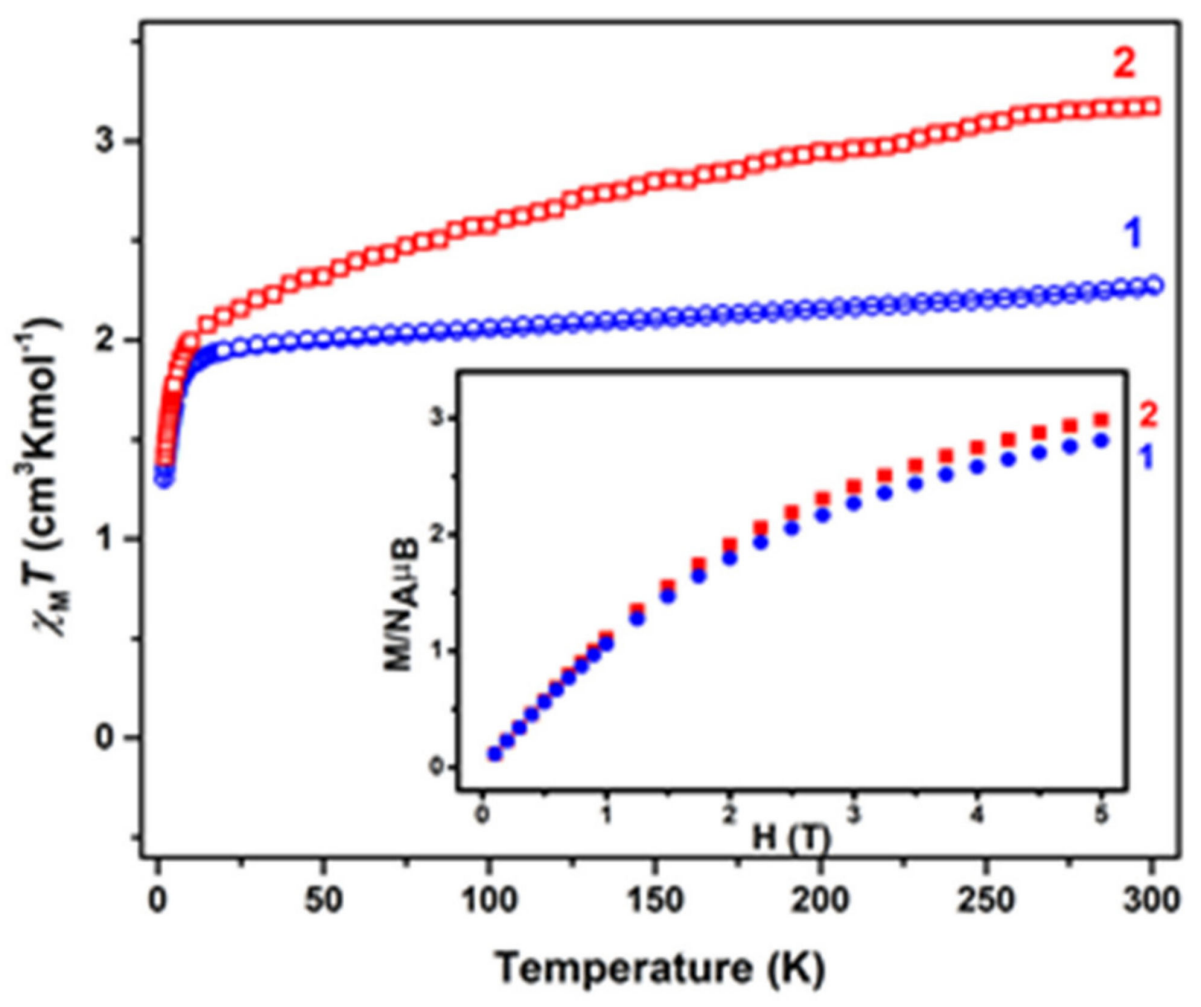
Plots of χ_M_*T* vs. *T* at 1000 Oe and *M* vs. *H* at 2 K (inset) for **1** (**blue**) and **2** (**red**). Solid line for **1** represents the best fit obtained with the magnetic model described in the text.

The χ_M_*T* product of **1** at room temperature is 2.27 cm^3^Kmol^−1^, which is larger than the calculated value of 2.09 cm^3^Kmol^−1^ for two Ni^II^ (*S* = 1, g = 2.0, χ_M_*T* = 2.0 cm^3^Kmol^−1^) and one Sm^III^ non-interacting ions (χ_M_*T* = 0.09 cm^3^Kmol^−1^). The value of χ_M_*T* product decreases slightly upon lowering the temperature, reaches a value of 1.94 cm^3^Kmol^−1^ at 20 K and then drops to 1.30 cm^3^Kmol^−1^ at 2 K (Figure 3). The susceptibility data were fitted considering (a) the intramolecular magnetic interaction between the two Ni^II^ ions, *J*, and (b) the magnetic anisotropy of the Ni^II^ ions, *D*, according to the spin Hamiltonian:

(2) $$H=-2JS_{Ni1}S_{Ni2}+2DS_{Ni}^{2}.$$

The best fit gave *J* = −0.02 cm^−1^, *D* = −7.67 cm^−1^ with g = 1.97 and TIP = 0.001 cm^3^mol^−1^. Taking into account the negligible value of *J*, which is in agreement with the very large intramolecular Ni···Ni distance, the fit of the χ_M_*T* vs. *T* data was repeated considering only the magnetic anisotropy of the Ni^II^ ions, and gave *D* = −8.07 cm^−1^ with g = 1.97 and TIP = 0.001 cm^3^mol^−1^. These values for the zero field splitting parameter *D* are extremely large for this type of complexes, suggesting that antiferromagnetic intramolecular interactions are present and affect the decrease of χ_M_*T* product at low temperatures. The χ_M_*T* vs. *T* data could be nicely fitted considering only the magnetic interaction between the Ni^II^ ions, leading to the value of *J* = −0.37 cm^−1^ with g = 1.97 and TIP = 0.001 cm^3^mol^−1^ (blue line in Figure 3).

The *χ*_M_*T* product of **2** at 300 K is 3.17 cm^3^Kmol^−1^, significantly higher than expected for two non-interacting Ni^II^ (S = 1, g = 2.0) ions and one Eu^III^ ion (S = 0). This behavior can be explained if we assume that the first excited states for the Eu^III^ ion are energetically close enough to the ground state so that can be thermally populated at 300 K. As the temperature decreases, the *χ*_M_*T* product of **2**, decreases to ~2 cm^3^Kmol^−1^ at 10 K (Figure 3). This is expected due to the progressive and final depopulation of the magnetic excited states of the Eu^III^ ions. Below 10 K, the *χ*_M_*T* product of **2** decreases to 1.41 cm^3^Kmol^−1^ at 2 K, which is close to the value of the *χ*_M_*T* product of **1** at 2 K, suggesting that both compounds exhibit similar ground states and that the Eu^III^ low-lying excited states in **2** are completely depopulated at 2 K.

Field dependent magnetization measurements were performed up to 5 T for **1** and **2** at 2 K; these are shown as insets in Figure 3. In both cases, the magnetization increases gradually in low magnetic fields and reaches 2.80 Nμ_B_ for **1** and 2.98 Nμ_B_ for **2** at 5 T, without reaching saturation.

**Figure 4:**
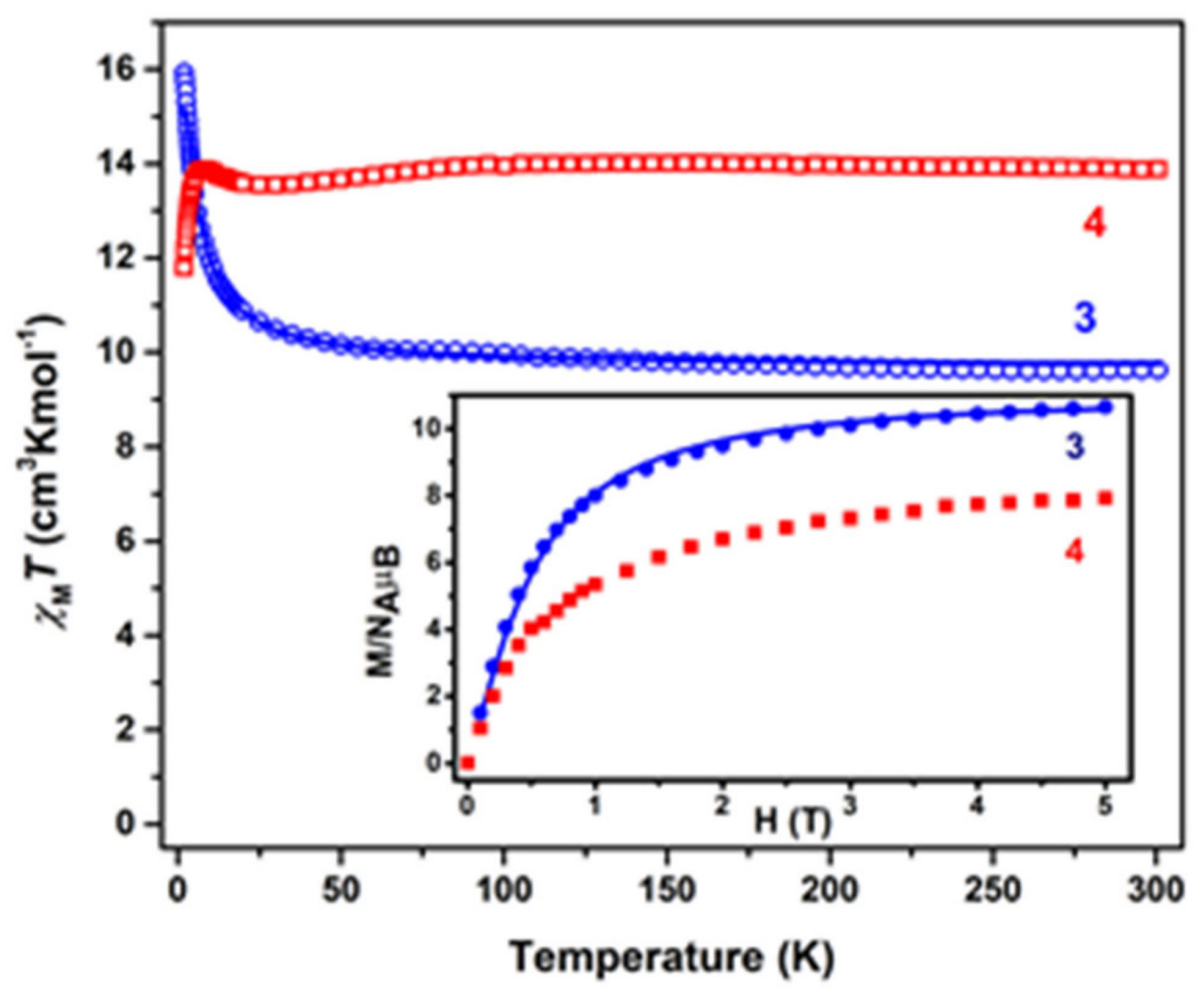
Plots of *χ*_M_*T* vs. *T* at 1000 Oe and *M* vs. *H* at 2 K (inset) for **3** (blue) and **4** (red). Solid lines for **3** represent the best fit obtained with the magnetic model described in the text.

The *χ*_M_*T* product of **3** at 300 K is 9.63 cm^3^Kmol^−1^ which is in very good agreement with the theoretic value (9.88 cm^3^Kmol^−1^) expected for two non-interacting Ni^II^ (*S* = 1, g = 2.0) and one Gd^III^ (*S* = 7/2, *J* = 7/2, g = 2.0) ions. Between 300 and 60 K, the *χ*_M_*T* product remains practically constant, and then increases slightly up to ~30 K; below that temperature the *χ*_M_*T* product increases rapidly and reaches the value of 15.92 cm^3^Kmol^−1^ at 2 K (Figure 4). The overall behavior is consistent with the presence of dominant ferromagnetic Ni···Gd interactions within the trinuclear complex. Magnetization measurements at 2 K show a rapid increase upon increasing of the magnetic field reaching a value of 10.64 Nμ_B_ at 5 T (Figure 4, inset) very close to the theoretical value for a *S* = 11/2 spin ground state corresponding to ferromagnetic coupling between two Ni^II^ and one Gd^III^ ions. Complex **3** is isomorphous to **4** as determined by unit cell measurements. For the latter, the molecular symmetry implies strictly linear metal arrangement and two equal Ni···Ln distances, which would require only one exchange parameter *J*_NiGd_. The magnetic susceptibility and isothermal *M*(*H*) data were fitted by using the spin Hamiltonian:

(3) $$H=-2J_{NiGd}(S_{Ni1}S_{Gd}+S_{Ni2}S_{Gd})+D(S_{Ni1}^{2}+S_{Ni2}^{2})$$

where *S*_Ni_ = 1 and *S*_Gd_ = 7/2 and *D* the magnetic anisotropy of the Ni^II^ ions. The best fit gave *J*_NiGd_ = +0.42 cm^−1^, *D* = +2.95 cm^−1^ with g_Ni_ = g_Gd_ = 1.98 (solid lines in Figure 4). These values agree with those found in other linear trinuclear Ni_2_Gd complexes [13,20].

**Figure 5:**
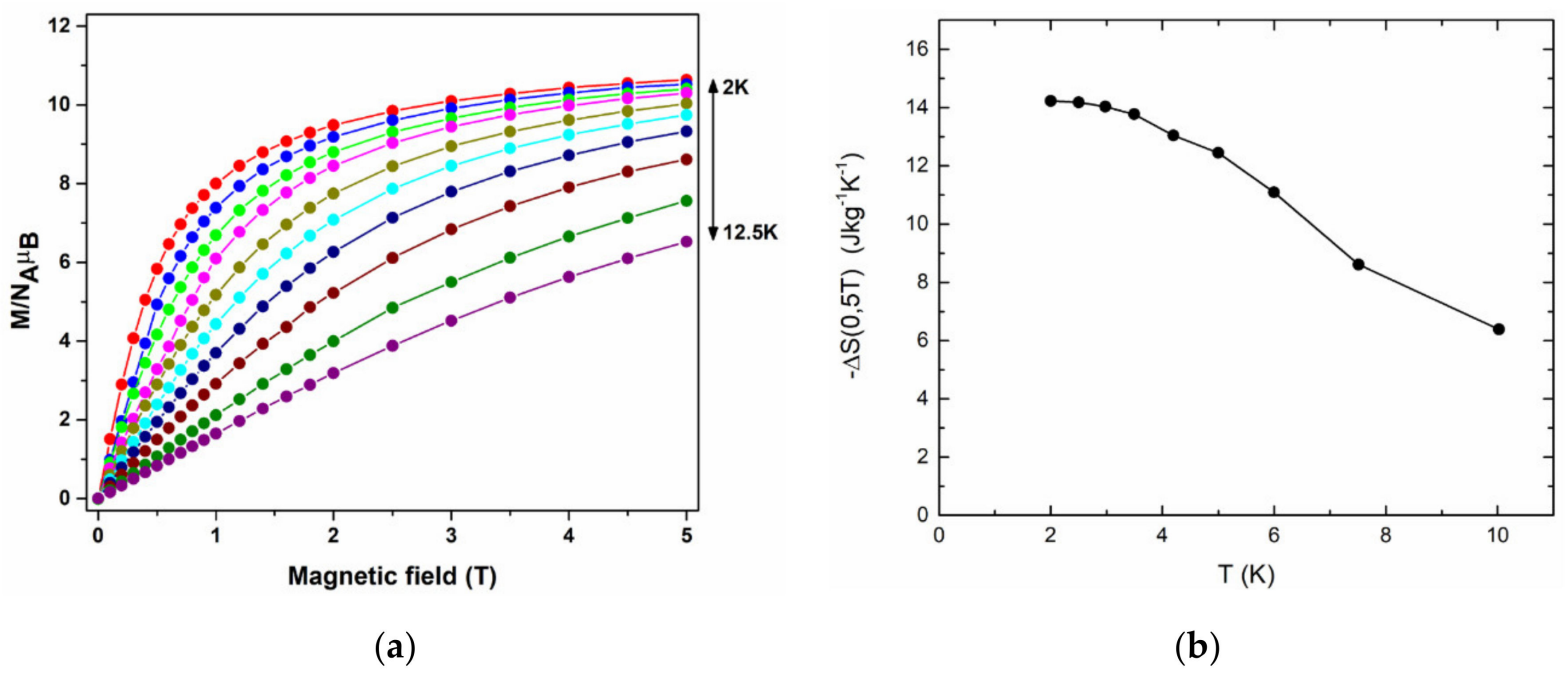
(**a**) Field dependent isothermal magnetization of **3** in the temperature range 2–12.5 K. (**b**) A plot of the maximum entropy change from 0 to 5 Tesla, against temperature for **3**, calculated from the Equation (5). Solid lines are guide to the eye.

The magnetocaloric effect of **3** was determined by isothermal magnetization measurements in the temperature range 2–12.5 K under applied magnetic fields up to 5 T (Figure 5). The magnetic entropy change can be obtained by using Maxwell’s relation

(4) $$\Delta S_{m}(T,H)=\overset{B_{f}}{\underset{B_{i}}{\int }}{\left[\frac{\partial M(T,H)}{\partial T}\right]}_{H}dB$$

where *B* is the applied magnetic induction in Tesla, *B*_i_ and *B*_f_ are the initial and final applied magnetic induction. A simple numerical approach can be used to obtain the experimental value of the entropy from the *M* vs. *H* curves using Equation (5)

(5) $$\left|\Delta S_{m}(T_{i})\right|={\left|\sum_{i}\frac{M_{i+1}-M_{i}}{T_{i+1}-T_{i}}\right|}_{H}\Delta B_{i}$$

where *M_i_* and *M_i_*_+1_ are the magnetization values measured in a field H at temperatures *T_i_* and *T_i_*_+1_, respectively.

The change of the magnetic entropy was calculated |*S*(0,5T)| = 14.2 Jkg^−1^K^−1^ at T = 2 K in agreement with the values found in the literature [17]. Moreover, the estimated value of the |*S*(0,5T)| is lower than the theoretically expected for three independent ions (two Ni^II^ *S* = 1 and one Gd^III^ S = 7/2), Δ*S* = 2*R*ln(3) + *R*ln(8) = 4.276*R* Jmol^−1^K^−1^ ≡ 35 Jmol^−1^K^−1^. With a molecular weight of 1.35 kgmol^−1^ for **3**, an entropy change of 26.33 Jkg^−1^K^−1^ is expected. This difference can be attributed to the presence of non-zero exchange interactions. Nevertheless, our results are comparable with the values found in the literature in a similar Ni_2_Gd cluster [17].

The *χ*_M_*T* product of **4** at 300 K is 13.88 cm^3^Kmol^−1^, which is close to the theoretical value of 13.81cm^3^Kmol^−1^, for two Ni^II^ (*S* = 1, g = 2.0) and one Tb^III^ (*S* = 3, *J* = 6, g = 3/2) non-interacting ions (Figure 4). The *χ*_M_*T* product of **4** remains practically constant in the temperature range 300–80 K and then decreases slightly to the value of 13.54 cm^3^Kmol^−1^ at 25 K. This behavior indicates dominant intramolecular antiferromagnetic interactions between the metal ions and/or thermal depopulation of the Tb^III^ excited states. Between 25 and 8 K, the *χ*_M_*T* product increases slightly to reach the value of 13.88 cm^3^Kmol^−1^ and then drops to 11.79 cm^3^Kmol^−1^ at 2 K. This behavior could be attributed to ferromagnetic interactions between the metal ions that could result in a high spin ground state.

The field dependence of the magnetization for **4** is shown in Figure 4 inset. The magnetization of **4** at 2 K reaches 7.92 Nμ_B_ at 5 T. The curve does not level out as magnetization does not saturate suggesting that ground spin state is not fully populated because other excited states remain populated to some extent.

## 3. Materials and Methods

### 3.1. General and Spectroscopic Measurements

All manipulations were performed under aerobic conditions using materials as received (Aldrich Co). All chemicals and solvents were of reagent grade. The ligand OH-C_6_H_4_-CH=NC(CH_2_OH)_3_, H_4_L was synthesized as described previously [57]. Elemental analysis for carbon, hydrogen, and nitrogen was performed on a Perkin Elmer 2400/*II* automatic analyzer. Infrared spectra were recorded as KBr pellets in the range 4000–400 cm^−1^ on a Bruker Equinox 55/S FT-IR spectrophotometer. Variable-temperature magnetic susceptibility measurements were carried out on polycrystalline samples of **1**–**4** by using a SQUID magnetometer (Quantum Design MPMS 5.5). Diamagnetic corrections were estimated from Pascal′s constants. The program PHI was used for simulations of the magnetic susceptibility data of **3** [58].

### 3.2. Compound Preparations

#### 3.2.1. General Synthetic Route for [Ni_2_Ln(H_3_L)_4_(O_2_CMe)_2_](NO_3_) (**1**–**4**)

Solid Ln(NO_3_)_3_·6H_2_O (0.25 mmol) was added under stirring to a yellow solution of H_4_L (1.00 mmol, 0.2252 g) in EtOH (20 mL). The solution was refluxed for three hours until solid Ni(O_2_CMe)_2_·4H_2_O (0.50 mmol, 0.1244 g) was added and continue refluxing for two more hours. The final green solution was layered with mixture of Et_2_O/*n*-hexane (1:1 *v*/*v*) to afford light green microcrystalline solid or X-ray quality single crystals after approximately two weeks. The material was filtered off and dried in vacuo.

##### 3.2.2. [Ni_2_Sm(H_3_L)_4_(O_2_CMe)_2_](NO_3_) (**1**)

Sm(NO_3_)_3_·6H_2_O (0.25 mmol, 0.1111 g), Yield: 0.069 g, ~20% based on Sm^III^. C_48_H_62_N_5_O_23_Ni_2_Sm requires C, 42.87; H, 4.65; N, 5.21%. Found: C, 42.78; H, 4.63; N, 5.19%. FT-IR (KBr pellets, cm^−1^): 3452(br), 2980(w), 2940(w), 2895(w), 1638(vs), 1600(m), 1556(s), 1473(m), 1442(m), 1384(vs), 1350(sh), 1317(sh), 1284(m), 1251(m), 1199(m), 1150(m), 1128(w), 1057(vs), 942(m), 919(w), 890(m), 857(w), 810(m), 764(m), 742(m), 675(m), 634(m), 610(w), 588(m), 541(w), 455(w).

##### 3.2.3. [Ni_2_Eu(H_3_L)_4_(O_2_CMe)_2_](NO_3_) (**2**)

Eu(NO_3_)_3_·6H_2_O (0.25 mmol, 0.1115 g), Yield: 0.046 g, ~14% based on Eu^III^. C_48_H_62_N_5_O_23_Ni_2_Eu requires C, 42.82; H, 4.64; N, 5.20%. Found: C, 42.73; H, 4.23; N, 5.18%. FT-IR (KBr pellets, cm^−1^): 3450(br), 2940(w), 2895(w), 1638(vs), 1599(m), 1556(s), 1473(m), 1444(m), 1384(vs), 1348(w), 1284(m), 1251(m), 1200(m), 1150(m), 1128(w), 1050(vs), 941(m), 890(m), 855(w), 811(m), 765(m), 742(m), 675(m), 633(m), 610(w), 587(m), 540(w), 455(w).

##### 3.2.4. [Ni_2_Gd(H_3_L)_4_(O_2_CMe)_2_](NO_3_) (**3**)

Gd(NO_3_)_3_·6H_2_O (0.25 mmol, 0.1128 g), Yield: 0.070 g, ~21% based on Gd^III^. C_48_H_62_N_5_O_23_Ni_2_Gd requires C, 42.65; H, 4.62; N, 5.18%. Found: C, 42.56; H, 4.60; N, 5.16%. FT-IR (KBr pellets, cm^−1^): 3437(br), 2940(w), 2855(w), 1637(vs), 1598(s), 1556(s), 1474(s), 1445(s), 1384(vs), 1340(w), 1317(w), 1286(vs), 1250(w), 1201(m), 1149(m), 1045(vs), 943(m), 889(m), 857(w), 813(m), 774(s), 742(m), 686(m), 634(m), 613(w), 584(m), 548(w), 517(w), 497(w), 475(w), 453(w).

##### 3.2.5. [Ni_2_Tb(H_3_L)_4_(O_2_CMe)_2_](NO3) (**4**)

Tb(NO_3_)_3_·6H_2_O (0.25 mmol, 0.1133 g), Yield: 0.047 g, ~14% based on Tb^III^. C_48_H_62_N_5_O_23_Ni_2_Tb requires C, 42.60; H, 4.62; N, 5.18%. Found: C, 42.51; H, 4.60; N, 5.16%. FT-IR (KBr pellets, cm^−1^): 3461(br), 2940(w), 2860(w), 1638(vs), 1600(s), 1556(s), 1474(s), 1446(s), 1384(vs), 1344(w), 1320(w), 1285(s), 1253(m), 1202(m), 1150(m), 1126(m), 1045(s), 986(w), 944(m), 890(m), 857(m), 810(m), 772(s), 742(m), 684(m), 633(m), 613(w), 586(w), 546(w), 519(w), 479(w), 453(w).

### 3.3. Single Crystal X-ray Crystallography

Crystals of **4**·4EtOH·4H_2_O (0.07 × 0.10 × 0.42 mm) were taken from the mother liquor and immediately cooled to −113 °C. Diffraction measurements were made on a Rigaku R-AXIS SPIDER Image Plate diffractometer using graphite monochromated Cu Kα radiation. Data collection (ω-scans) and processing (cell refinement, data reduction and empirical absorption correction) were performed using the CrystalClear program package [59]. The structure was solved by direct methods using SHELXS v.2013/1 and refined by full-matrix least-squares techniques on F^2^ with SHELXL ver2014/6 [60,61] (see Appendix A). Important crystallographic and refinement data are listed in Table 3. Further experimental crystallographic details for **4**·4EtOH·4H_2_O: 2*θ*_max_ = 130°; reflections collected/unique/used, 14249/3026 [R_int_ = 0.0671]/3026; 180 parameters refined; (Δ/*σ*)_max_ = 0.002; (Δ*ρ*)_max_/(Δ*ρ*)_min_ = 3.761/−1.538 e/Å^3^; *R*1/w*R*2 (for all data), 0.1016/0.2418. Hydrogen atoms were either located by difference maps and were refined isotropically or were introduced at calculated positions as riding on bonded atoms. All non-hydrogen atoms were refined anisotropically. Plots of the structure were drawn using the Diamond 3 program package [62].

## 4. Conclusions

We have demonstrated the preparation of the trinuclear complexes of general formula [Ni_2_Ln(H_3_L)_4_(O_2_CMe)_2_](NO_3_), Ln = Sm (**1**), Eu (**2**), Gd (**3**), Tb (**4**), which contain the monoanion of the tetradentate Schiff base ligand H_4_L: *o*-OH-C_6_H_4_-CH=N-C(CH_2_OH)_3_. The crystal structure of **4** revealed the presence of complex cations and nitrate anions. The complex cations contain two terminal Ni^II^ ions and one central Tb^III^ ion in linear arrangement. Both Ni^II^ ions present distorted octahedral geometry and are bound to two H_3_L^−^ ligands through their phenolato oxygen, the imino nitrogen atoms and one of the protonated alkoxo groups. The Tb^III^ ion is coordinated to four phenolato oxygen atoms from the four ligands, and four carboxylato oxygen atoms from two acetates which are bound in the bidentate chelate mode. The use of Continuous Shape Measures approach (CShM) revealed that the coordination polyhedron around the terbium ion in **4** is square antiprism, SAPR-8. The pendant alkoxide arms of the ligands participate in an extensive network of hydrogen bonds. The final architecture of the lattice structure consists of two interpenetrating 3D diamond lattices which host the NO_3_^−^ counteranions and the lattice solvents. The identity of **3** was confirmed by unit cell determination and it was found isomorphous to complex **4**. The chemical identity and similarity of **1**–**4** were confirmed by infrared spectroscopy. The magnetic study of these complexes demonstrated the nature of the magnetic exchange between the metal ions in **1**–**4**. The magnetic susceptibility measurements revealed weak antiferromagnetic coupling between the Ni^II^ ions in **1**. The contribution of the magnetic anisotropy of the Ni^II^ ions (*S* = 1) during the fit of the susceptibility data of **1** led to unrealistic high values for the parameter *D* and was not taken into account. The significantly high value of the *χ*_M_*T* product of **2** at 300 K is consistent with the presence of first excited states which are sufficiently low in energy and are thermally populated at r.t. The experimental χ_M_*T* vs. *T* curve for **4** indicates the presence of intramolecular antiferromagnetic interactions between the metal ions; the decrease of the *χ*_M_*T* products at low temperatures are consistent with the thermal depopulation of the Tb^III^ excited states. The magnetic study of **3** revealed dominant ferromagnetic interactions between the Ni^II^ and Gd^III^ ions with *J*_NiGd_ = +0.42 cm^−1^, *D* = +2.95 cm^−1^ (g_Ni_ = g_Gd_ = 1.98), resulting in *S* = 11/2 spin ground state. The change of the magnetic entropy of **3** was calculated |Δ*S*(0,5T)| = 14.2 Jkg^−1^K^−1^ at T = 2 K in agreement with the values found in the literature.

Further work is in progress in our lab with other lanthanides in order to systematically delve into the magnetic properties of these complexes.

## Figures and Tables

**Table 1:** Selected bond distances (Å) in **4**·4EtOH·4H_2_O.

**Tb1—O1**	2.323 (4)	**Ni—N**	2.038 (6)
**Tb1—O1′**	2.323 (4)	**Ni—N‴**	2.038 (6)
**Tb1—O1″**	2.323 (4)	**Ni—O1‴**	2.074 (5)
**Tb1—O1‴**	2.323 (4)	**Ni—O1**	2.074 (5)
**Tb1—O5**	2.469 (4)	**Ni—O3**	2.085 (6)
**Tb1—O5″**	2.469 (4)	**Ni—O3‴**	2.085 (6)
**Tb1—O5′**	2.469 (4)	**Tb1—O5‴**	2.469 (4)

Symmetry operation: (′) 1.5-*x*, 0.5-*y*, *z*; (″) *x*, 0.5-*y*, 1.5-*z*; (‴) 1.5-*x*, *y*, 1.5-*z*.

**Table 2:** Hydrogen bonds in **4**·4EtOH·4H_2_O.

Interaction	D···A (Å)	H···A (Å)	D-H···A (^°^)	Symmetry Operation
O(2)-H(2O)···O(5)	2.711	1.884	172.6	x, y, z
O(3)-H(3O)···O(1E)	2.611	1.873	152.8	0.5 + x, −y, 0.5 − z
O(4)-H(4O)···O(2)	2.748	1.914	171.7	2 − x, −y, 1 − z

**Table 3:** Crystallographic data for **4**·4EtOH·4H_2_O.

	4·4EtOH·4H_2_O
Formula	C_56_H_94_N_5_Ni_2_O_31_Tb
*Fw*	1609.70
Space group (system)	*P*nnn (orthorhombic)
*a* (Å)	10.0634(2)
*b* (Å)	18.2467(3)
*c* (Å)	19.8388(4)
*V* (Å^3^)	3642.88(12)
*Z*	2
*T* (^o^C)	−113
Radiation	Cu Kα 1.54178
*ρ*_calcd_, g cm^−3^	1.468
*μ*, mm^−1^	6.017
Reflections with *I > 2σ(I)*	2053
*R*_1_^a^	0.0781
w*R*_2_^a^	0.2045

^a^ w = 1/[*σ*^2^(*F*_o_^2^) + (*αP*)^2^ + *bP*] and *P* = (max *F*_o_^2^,0) + 2*F*_c_^2^)/3, *R*_1_ = ∑(|*F*_o_| − |*F*_c_|)/∑(|*F*_o_|) and w*R*_2_ = {∑[w(*F*o^2^ − *F*c^2^)^2^]/∑[w(*F*_o_^2^)^2^]}^1/2^.

## Supplementary Materials

The following are available online. Figure S1: The FT-IR spectra of complexes **1**–**4**. Figure S2: The characteristic interlocked adamantane units of two independent interpenetrating diamondlike lattices observed in the structure of compound **4**.

## Appendix A

CCDC 1994669 contains the supplementary crystallographic data for this paper. These data can be obtained free of charge from The Cambridge Crystallographic Data Centre via www.ccdc.cam.ac.uk/data_request/cif.
