# Supplementary material for: Trinuclear NiII-LnIII-NiII Complexes with Schiff Base Ligands: Synthesis, Structure, and Magnetic Properties
Source: Molecules. 2020 May 12;25(10):2280. doi: 10.3390/molecules25102280 (PMC7288099; doi:10.3390/molecules25102280)
Supplement: Supplementary file 1 [file molecules-25-02280-s001.pdf]

Supplementary material

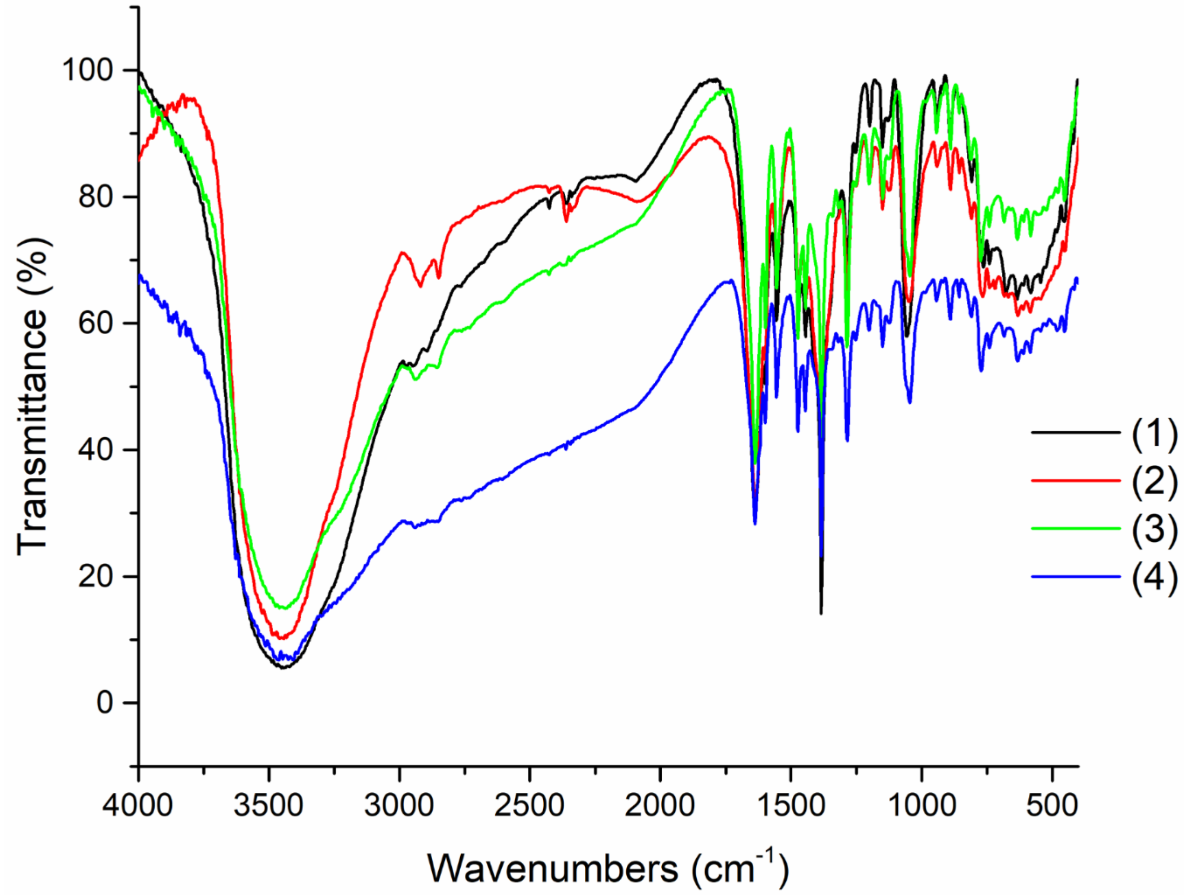

Figure S1. The FT-IR spectra of complexes **1-4**.

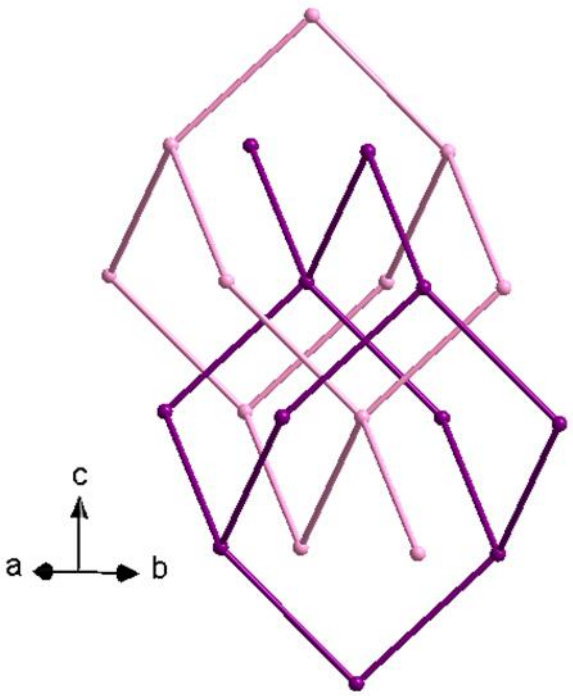

Figure S1. The characteristic interpenetrating adamantane units of the two independent diamondlike lattices [49] in the structure of compound **4**.
